# Investigation of the Effect of Nanoclay on Thermal and Dynamic Mechanical Properties of Poly(*N*‑vinyl-2-pyrrolidone)/Dextran/Poly(ethylene glycol)-Based Nanocomposites

**DOI:** 10.1021/acsomega.6c03988

**Published:** 2026-09-13

**Authors:** Serap Kavlak

**Affiliations:** Faculty of Science, Department of Chemistry, 37515 Hacettepe University, 06800 Ankara, Türkiye

## Abstract

In this work, the effect of nanoclay content on the thermal and dynamic mechanical properties of the ternary polymer blend-based nanocomposites was investigated. The synergistic effect of combining poly­(*N*-vinyl-2-pyrrolidone) (PVP), dextran (DEX), and poly­(ethylene glycol) (PEG), along with the addition of nanoclay, can lead to the formation of new nanostructures with improved properties. Based on this, nanocomposites were successfully prepared using the solution mixing method. Structural and morphological characteristics of the prepared nanocomposites were comprehensively evaluated by Fourier transform infrared (FTIR) spectroscopy, X-ray diffraction (XRD), X-ray photoelectron spectroscopy (XPS), and transmission electron microscopy (TEM) techniques. Intercalation of the polymer blend between the silicate layers was determined by XRD analysis. TEM analyses confirmed highly intercalated nanocomposite structures. The thermal and dynamic mechanical behavior of the ternary blend and its nanocomposites was investigated by thermogravimetric analysis (TGA) and dynamic mechanical analysis (DMA). The nanocomposites exhibited enhanced thermal stability compared to the polymer blend, as evidenced by residue percentages of 8.3% for the blend and 16.1 and 17.4% for the nanocomposites containing 5 and 10% nanoclay at 600 °C, respectively. For the ternary blend, the onset temperature observed in the storage modulus was 47.8 °C, while for nanocomposites containing 5 and 10% nanoclay, this temperature was 51.6 and 52.4 °C, respectively; the differences are slightly greater at temperatures above ∼200 °C. These functional nanocomposites, prepared through the synergy of multiple materials and containing biocompatible polymers, have been developed as potential candidate nanomaterials for use in various fields requiring high-performance materials.

## Graphical abstract

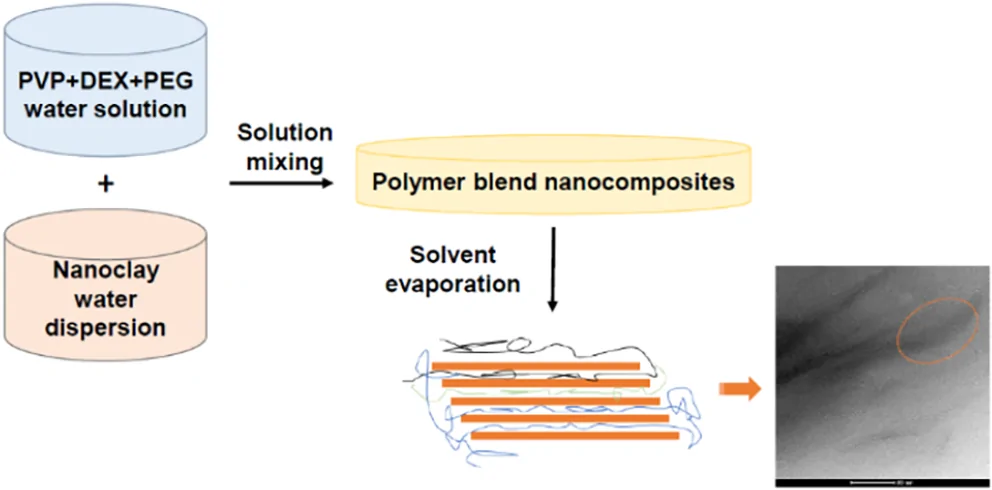

## 1 Introduction

Nowadays, to meet the increasing demand for new high-performance materials, improving material properties by taking advantage of synergistic effects created by combining multiple materials is of great importance.^1,2^ Polymeric materials with desired properties can be easily and cost-effectively prepared by blending different polymers. In this way, new properties can be obtained or existing properties can be improved for polymeric materials.^3,4^ Combining more than one polymer with different functional groups and creating intermolecular interactions between these units play a significant role in developing new polymer blends with desired properties.^5^ Studies and investigations on polymer blend-based materials have gained importance due to their applications in bioengineering, industry, energy storage, and packaging.^6–10^ In this context, the thermomechanical and rheological properties, as well as the degradation behavior, of polymer blends also become important in determining their usage limits.^11^

Polymer blend-based layered silicate nanocomposites have attracted the interest of scientists and industrial manufacturers due to their enhanced properties in the presence of silicate particles and other nanofillers, cost-effective and easy preparation methods, and a wide range of applications.^12–14^ With advancements in nanotechnology and the expansion of application areas for nanostructured hybrid materials, the production of functional polymer-based nanocomposites has gained particular importance.^15^ In addition to the synergistic effect obtained with polymer blend, the addition of nanoclay materials as a nanofiller can also cause improving effects such as reforming the morphology of the polymeric material and mechanical improvement.^16,17^ In particular, high-performance biocompatible and biodegradable materials can be obtained by integrating nanofillers into the polymer structure.^18^ Various layered silicates can be added as nanofillers to polymers in order to increase the thermal, dielectric, and mechanical features of polymeric materials and to enhance dimensional stability, and thus, polymer blend–clay nanocomposite structures with desired properties can be obtained.^19,20^ Montmorillonite (MMT) is one of the important clays due to its natural origin, which is a key feature for sustainability, as well as its cation exchange capacity, barrier property, heat resistance, and water sorption properties.^21^

Polymer-based clay nanocomposites can be prepared through mainly the in situ intercalative polymerization method,^15^ in situ template synthesis, and melt intercalation method.^22^ Apart from these, another method that stands out due to its easy preparation feature is the intercalation of the polymer from the solution or the solution mixing technique.^23^ In solution mixing method, there is a solvent system in which a polymer dissolves and the silicate layers of the clay swell. The layered silicate swells in a solvent; then, the polymer solution and clay dispersion are combined at the stage in which the polymer chains intercalate and displace the solvent in the silicate interlayer.^24^ Intercalated or exfoliated polymer-based clay nanocomposites are obtained depending on the compatibility/interaction of the clay and polymer with the solution mixing method.^25^

Extensive research has been performed on clay nanocomposites with various polymer blends up to now, but especially, the improvement of their thermal and mechanical properties is still ongoing.^26^ Nanocomposite materials based on a blend of biocompatible and/or biodegradable polymers attract special attention due to their functional groups.^27^ The dielectric/electrical and structural properties of binary hydrophilic polymer blends containing poly­(vinyl alcohol)–poly­(vinylpyrrolidone), poly­(vinyl alcohol)–poly­(ethylene glycol) (PEG), poly­(vinyl alcohol)–poly­(ethylene oxide), and MMT clay nanomaterials were investigated.^28,29^ Compatibility and synergistic effects in structural and thermal properties in poly­(ethylene glycol)/poly­(vinylpyrrolidone) blend/sepiolite clay-containing nanocomposites were identified.^30^ Multifunctional colloidal nanofiber nanocomposites of poly­(*N*-vinyl-2-pyrrolidone) (PVP)/dextran (DEX) blends with ODA-MMT were fabricated by electrospinning in the presence of folic acid, and the chemical/physical structures, morphology, and electrical properties of poly­(*N*-vinyl-2-pyrrolidone)/dextran/ODA-MMT nanocomposite materials were investigated for biomedical applications by Rzayev et al.^31^ Poly­(acrylic acid)/starch and poly­(acrylic acid)/dextran nanocomposites were prepared by reinforcing with nanoclays for packaging applications.^32,33^

The main objective of this work is to investigate and evaluate the effect of an organically modified MMT nanoclay on the thermal and dynamic mechanical behaviors of poly­(*N*-vinyl-2-pyrrolidone)/dextran/poly­(ethylene glycol) (PVP/DEX/PEG)-based nanocomposites. The novelty of the present work lies in evaluating the effect of nanoclay content on thermal and dynamic mechanical properties in a system combining the synergy of a ternary polymer blend with nanoclay, using comprehensive characterization techniques; these highlight that existing polymer properties can be improved at the nanoscale and that thermal stability and dynamic mechanical properties can be improved by leveraging the synergy of the ternary polymer blend. In this regard, most studies in the literature on polymer blend/clay materials have focused on nanocomposites based on binary polymer blends, but there are few studies of nanocomposites obtained from ternary polymer blends. Therefore, in this study, due to the existing properties and application potentials of the selected polymers, the synergy created by the combined use of the ternary polymer blend has become even more significant with the addition of nanoadditive. Characterizations performed with comprehensive methods also explain the relationships between the structures and properties of these nanocomposites, contributing to the gap in the literature in terms of scope, method, and approaches, and optimization results obtained, by both producing ternary polymer blend-based nanocomposites with a facile method and investigating their thermal and dynamic mechanical properties in particular. Compared to the existing literature, the results of this study aim to offer new opportunities for developing new functional nanomaterials with enhanced thermal stability and comprehensive characterization. Furthermore, the findings will offer a simpler and efficient approach for preparing functional materials with improved properties using ternary polymer blends and nanoadditive content compared to other complex methods. With the increasing applications of the nanomaterials mentioned above, the cost-effective preparation of nanomaterials containing functional groups using easier methods such as solution mixing, as used in this study, has gained importance compared to more complex methods such as in situ copolymerization, chemical modification, or grafting, which are frequently seen in the literature.^15,32^ For this purpose, the motivation of this study is to prepare nanomaterials using a facile method by utilizing the synergy created by polymer blending with polymers containing functional groups and to investigate the effect of nanoadditives. Based on this, functional group-containing ternary polymer blend/nanoclay nanocomposites were prepared through a cost-effective solution mixing technique using PVP/DEX/PEG and organically modified MMT nanoclay. The effect of nanoclay composition on the nanocomposite structure and morphology was characterized by spectroscopic and microscopic analysis, while thermal stability and dynamic mechanical properties were investigated in detail by using thermogravimetric and dynamic mechanical analysis (DMA) techniques.

## 2 Experimental Section

### 2.1 Materials

The dextran polymer (DEX, molecular weight: 40,000, Sigma-Aldrich), poly­(*N*-vinyl-2-pyrrolidone) (PVP, molecular weight: 44,000, BDH), and poly­(ethylene glycol) (PEG, molecular weight: 8000, Aldrich) polymers were laboratory grade and used directly without purification. Nanomer (1.44P) nanoclay, containing dimethyl dialkyl amine (35–45 wt %) montmorillonite (MMT), was a product of Nanocor Inc. The water used in preparing the solutions was double-distilled.

### 2.2 Preparation of Ternary Polymer Blend/Nanoclay Nanocomposites

Ternary polymer blend/nanoclay nanocomposites were prepared by the solution mixing method. Nanocomposites were formulated by mixing the polymer PVP/DEX/PEG ternary system (45/45/10 weight ratio) and nanoclay at 0, 5, and 10 wt % based on polymer blends as nanofillers in water solutions (Table 1). In the preparation of nanocomposites, polymer molecular weights that could provide certain desired properties were selected. 5 and 10 wt % nanoclay percentages were chosen because previous studies have shown that nanoclay percentages below 10 wt % can improve certain properties of polymers.^34,35^ To prepare these materials, an aqueous solution of each polymer was first prepared by dissolving in double-distilled water overnight. Afterward, aqueous blend solutions were obtained by mixing the polymer solutions on the magnetic stirrer at the rate of 100 rpm after 1 h. Since previous studies have shown that mild conditions are sufficient for mixing these polymers, a relatively short mixing time and low mixing rate were chosen. Meanwhile, certain amounts of nanoclay were dispersed vigorously in double-distilled water on the magnetic stirrer at 1000 rpm for 3 h to ensure homogenization. After that, the prepared nanoclay dispersions were added to polymer blend mixtures under the same conditions. Subsequently, polymer blend/nanoclay mixtures were magnetically stirred at the rate of 500 rpm for 3 h in ambient conditions to obtain a homogeneous mixture. All the dispersions were then poured into the petri dishes, and all the polymer/nanoclay nanocomposites were obtained by solution casting after water evaporation. Finally, the polymer blend and the nanostructured materials were dried under vacuum at 50 °C. All the samples were kept under dry conditions and used directly in characterization.

**Table 1:** Sample Codes and Formulation of the Polymer Blend/Nanoclay Samples

material code	material	nanoclay (wt % based on the blend)
Blend/Nanoclay-0	PVP/DEX/PEG	0
Blend/Nanoclay-5	PVP/DEX/PEG/nanoclay	5
Blend/Nanoclay-10	PVP/DEX/PEG/nanoclay	10

### 2.3 Characterizations

#### 2.3.1 Fourier Transform Infrared (FTIR) Analysis

FTIR spectra of the ternary polymer blend and nanomaterials were taken by a PerkinElmer 400 FTIR spectrometer using an attenuated total reflectance (ATR) imaging accessory from 4000 to 400 cm^–1^ (16 scans, 4 cm^–1^ resolution).

#### 2.3.2 X-ray Diffraction (XRD) Analysis

XRD analysis of the powder samples was completed by a Rigaku MF X-ray diffractometer. The XRD diffractograms were measured in the 2θ range of 2–60°, with a scanning rate of 1° min^–1^ (Cu Kα, 15 mA, 40 kV). The *d*-spacing was calculated by Bragg’s law (*n*λ = 2*d* sin θ), where *d* is the interplanar spacing, n is an integer representing the order of reflection, λ is the wavelength of the X-rays, and θ is the angle of incidence.

#### 2.3.3 X-ray Photoelectron Spectroscopy (XPS)

XPS measurements of the samples were performed with a PHI 5000 VersaProbe spectrometer equipped with an Al monochromatic X-ray anode. The photoemission take-off angle was 45.0°, and the analyzer pass energy was 187.85 eV. Binding energy was varied between 0 and 1250 eV.

#### 2.3.4 Transmission Electron Microscopy (TEM)

TEM images of the nanocomposites were recorded with an FEI Tecnai G2 Spirit BioTwin high-contrast transmission electron microscope (CTEM) (accelerating voltage/electron source: 120 kV/LaB_6_). For the analysis, samples were diluted with ethanol and ultrasonicated, and a drop of the suspension was placed on a carbon-coated copper grid and left to dry.

#### 2.3.5 Thermogravimetric Analysis (TGA)

TGA of the ternary polymer blend and its nanocomposites was performed using a Perkin Elmer STA 6000 thermogravimetric analyzer. The samples were heated from ambient temperature to 600 °C at 10 °C min^–1^ heating rate under a nitrogen flow.

#### 2.3.6 Dynamic Mechanical Analysis (DMA)

DMA of the ternary polymer blend and ternary polymer blend-based nanocomposites was conducted using a TA Instruments DMA Q800 analyzer. Since the prepared polymer blend and nanocomposite samples were in the powder form, mixtures were prepared with aluminum oxide and then analyzed.^36^ The prepared samples and aluminum oxide were mixed (50:50 wt %) and they were loaded to the dual cantilever of the analyzer by a powder holder, which consists of upper and lower trays made of stainless steel. Temperature dependence of the ternary polymer blend and its nanocomposites was recorded at 1 Hz frequency and at 3 °C min^–1^ heating rate from ambient temperature to 245 °C.

## 3 Results and Discussion

### 3.1 Structure and Morphology

Structural properties of the ternary polymer blend and nanomaterials, especially interaction with the nanoclay and ternary polymer system, were investigated by FTIR spectroscopy. FTIR spectra of the PVP/DEX/PEG blend and its nanocomposites containing nanoclay are shown in Figure 1. The FTIR spectrum of the pristine PVP/DEX/PEG blend (Blend/Nanoclay-0) exhibited main absorption bands of the polymer units. A broad peak was seen at 3402 cm^–1^ responses for O–H stretching, and the strong peak observed at 2884 cm^–1^ corresponds to the asymmetric C–H stretching of CH_2_ in polymers. C=O stretching vibration peaks due to the carbonyl groups of PVP were observed at 1652 cm^–1^.^31^ C–H scissoring and C–H aliphatic bending vibrations were observed at 1463–1422 cm^–1^. CH_2_ wagging and C–N stretching appeared at 1281 cm^–1^.^37^ A sharp stretching vibration of C–O–C appeared at 1100 cm^–1^.^38,39^ CH_2_ in-plane bending was seen at 842 cm^–1^.^37^

**Figure 1:**
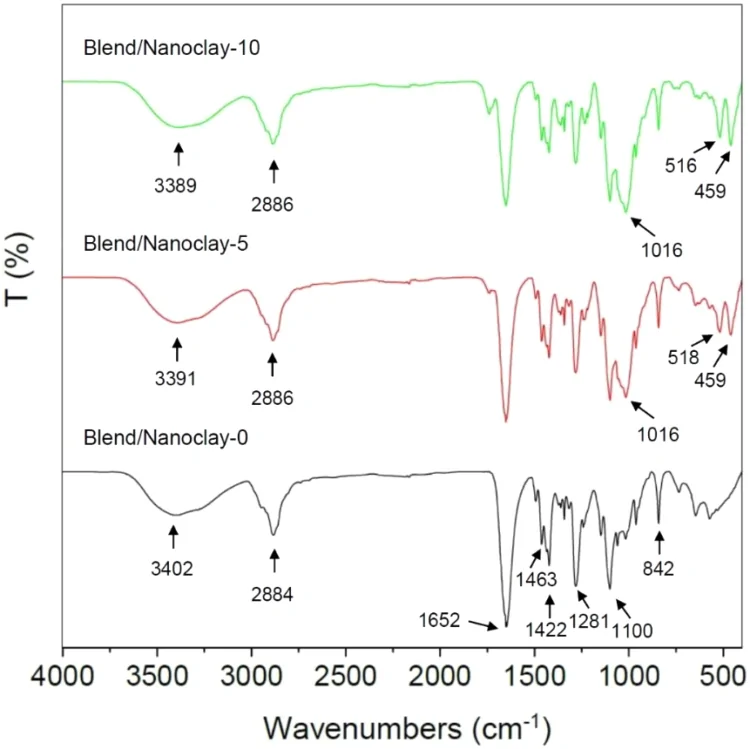
ATR-FTIR spectra of the materials.

When the FTIR spectra of the polymer blend/nanoclay nanocomposites (Figure 1) were examined, the bands of specific groups of nanoclay were observed along with the bands of functional groups of the polymers. Characteristic bands of the polymer blend have also been observed in the spectra of the nanomaterials. With the insertion of nanoclay to the polymer matrix, the bands seen in the spectra of the nanocomposites, different from the spectrum of the polymer blend, can be listed as follows: A characteristic O–H band of adsorbed water (free and in the interlayer) on the nanoclay surface and N–H stretching due to the presence of primary amine of the modified nanoclay appeared at ∼3600 cm^–1^ with a broad peak and a lower shoulder.^40^ The strong peak observed at 2886 cm^–1^ corresponds to the C–H bonds of alkyl groups due to the alkyl amine chain resulting from the modification of the nanoclay and polymer backbone.^15^ Another distinctive peak observed at 1016 cm^–1^ in the spectra of the nanocomposites comes from strong Si–O stretching bands resulting from the insertion of nanoclay to the nanocomposite structure, and the intensity of the band increased as the percentage of nanoclays increased from 5 to 10%. Al–O–Si and Si–O–Si bending and Al–OH vibration were also observed at 518, 516, and 459 cm^–1^ ^41,42^ in the spectra of the ternary polymer blend-based nanocomposites, respectively, because of the intercalation in the presence of nanoclay. These bands in the prepared ternary polymer blend-based nanocomposites demonstrated that the expected interactions between the ternary polymer blend and nanoclay occurred. The hydrogen bond interactions expected to occur between the polymer blend and the nanoclay can be determined by changes in the vibrational bands of the O–H groups. These interactions, and the changes observed in the FTIR spectrum, can provide information about the molecular-level interactions of the polymer blend and its interaction with the nanoclay. A broad band centered at 3402 cm^–1^ due to the O–H stretching was observed in the polymer blend, while bands centered at 3391 and 3389 cm^–1^ were detected in the Blend/Nanoclay-5 and Blend/Nanoclay-10 samples, respectively. In addition, the broadening of the O–H band toward lower wavenumbers with the addition of nanoclay, due to the presence of multiple free and intermolecular hydrogen-bonded O–H groups, also indicates that the interaction between the polymer blend and nanoclays occurs via hydrogen bonding.

The structure and the morphology of the prepared polymer blend-based layered silicate nanocomposites were identified by XRD, XPS, and TEM techniques. Representative XRD diffraction patterns of the ternary blend and ternary blend-based nanocomposites for the two different amounts of nanoclay are shown in Figure 2. XRD diffractograms provide information about the formation and structures of the nanocomposites by determining the *d*-spacing between the silicate layers and position of the corresponding response obtained from XRD diffractograms.^43^ The XRD pattern of the pristine polymer blend (Blend/Nanoclay-0) shows semicrystalline character with responses at 2θ = 10.9, 19.1° (sharp), 23.2° (sharp), 26.8, 29.4, and 36.2° (Figure 2). This semicrystalline character originates from the structure of PEG. The observed sharp responses are due to the presence of PEG and are consistent with the literature.^44^ It is known from the literature that PVP and dextran show a highly amorphous character, with main broad diffraction peaks at approximately 2θ = 11 and 22° for PVP and 2θ = 17° for dextran.^38,45,46^

**Figure 2:**
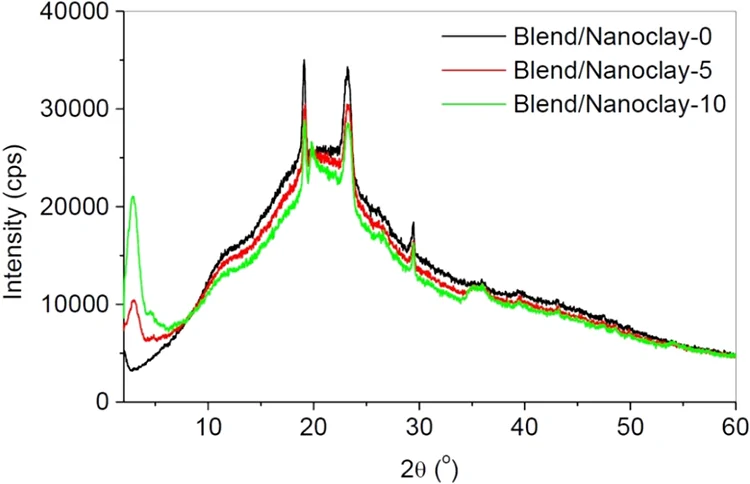
XRD diffractograms of the materials (2θ = 2–60°).

Representative XRD patterns of Blend/Nanoclay-5 and Blend/Nanoclay-10 nanocomposites (Figure 2) showed nearly identical responses accompanying the pristine polymer blend with a semicrystalline character. However, some important differences were also observed due to the formation of the nanocomposites in the presence of nanoclay. The intensities of the two main sharp peaks at 2θ = 19.1 and 23.2° regularly decreased with the addition of organically modified nanoclay incorporated into the structure due to the formation of the nanostructures. The main difference observed in the diffractograms of the nanocomposites was the responses between 2θ = 2 and 10° regions, which were not seen in the pristine polymer blend. The responses observed in the 2θ = 2–10° region was selected and studied in detail to examine the formation of nanocomposites (Figure 3). Tettenhorst and Roberson^47^ reported that MMT has large repeat distances and consequently basal spacing formed at small diffraction angles due to its complexation with organic species. In addition, they also explained that basal spacing shifts are more evident at lower diffraction angles. There is no response in the diffractogram of the pristine polymer blend (Blend/Nanoclay-0) at the 2θ = 2–10° region. However, XRD patterns of the two nanocomposites showed peaks at this interval. The XRD pattern of Blend/Nanoclay-5 showed a diffraction peak at 2θ = 2.95°, corresponding to a *d*-spacing of 29.9 Å. Diffraction peaks in the pattern of Blend/Nanoclay-10 were observed at 2θ = 2.92 and 4.53° with *d*-spacing values 30.3 and 19.5 Å, respectively (Table 2). The *d*-spacing values of this pristine nanoclay were reported previously as 2.46 nm at 2θ = 3.60° and 1.23 nm at 2θ = 7.18°.^48^ The weak response value is consistent with analogous hydrated clay structures reported in the literature. Due to modification with a surfactant, the characteristic response shifts to a lower angle, and a significant increase in interlayer distance is observed.^49^

**Figure 3:**
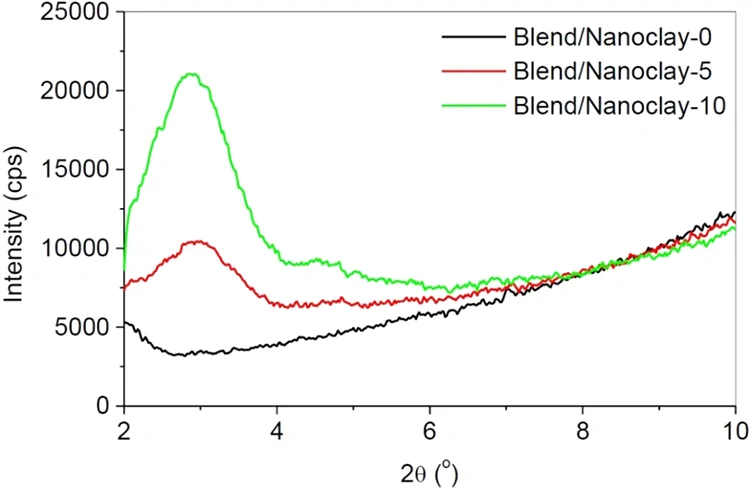
XRD diffractograms of the materials (2θ = 2–10°).

**Table 2:** XRD Results of the Prepared Materials (2θ = 2–10°)

materials	2θ (°)		*d*-spacing (Å)		Δ*d*-spacing (Å)		RI (%)	
Blend/Nanoclay-0								
Blend/Nanoclay-5	2.95		29.9		5.3		21.5	
Blend/Nanoclay-10	2.92	4.53	30.3	19.5	5.7	7.2	23.2	58.5

A relative intercalation (RI) can be computed as a percentage increase of the *d*-spacing value using the following eq 1 ^50^

1 $$RI=\left(\frac{d-d_{0}}{d_{0}}\right)\times 100$$

where *d*_0_ and *d* represent the *d*-spacing of the nanoclay and nanoclay in the ternary blend-based nanocomposites, respectively. RI in the Blend/Nanoclay-10 (RI = 23.2%) was higher than that of the Blend/Nanoclay-5 (RI = 21.5%) for the first response accompanied by an intercalated structure. RI for the second response of the Blend/Nanoclay-10 was calculated as 58.5% and it can be said that nanocomposites have an intercalated structure. The results showed that polymeric chains were intercalated between silicate layers. However, no second response was observed in the diffractogram of the Blend/Nanoclay-5. Since this response observed in nanoclay^48^ was not observed in the nanocomposite, it can be said that the small unmodified portion of the nanoclay contains a combination of the intercalated structure as well as a few partially exfoliated structures in the ternary blend-based nanocomposite. Studies in the literature also show that, for example, the addition of PVP to a poly­(vinyl alcohol)/Na^+^MMT matrix similarly results in the formation of exfoliated and highly intercalated PVA/PVP/Na^+^MMT nanocomposites.^51^

Another point observed in the XRD diffractograms of nanocomposites at the 2θ = 2–10° region is that the intensity of the responses observed in the nanocomposites increases with an increasing amount of added nanoclay.^52^ As a result of the XRD analysis, it was seen that the peaks shifted to the lower region and *d*-spacing in the silicate layers of the nanocomposites increased due to the increase in nanoclay loading and the intercalation of the ternary blend between silicate layers. Based on these results, it can be said that polar groups in the polymer blend, capable of containing associative-type interactions or hydrogen bonding, give rise to intercalation.^53^

XPS analysis is a surface-sensitive method and gives information about the elemental and chemical composition of the material due to the peak positions by measuring the binding energies of the elements in the structure. It was used to elucidate the structures of nanocomposites by examining their elemental composition and, consequently, the incorporation of silicate layers into the structure in order to support the FTIR, XRD, and TEM results. XPS survey spectra of the nanocomposites are shown in Figure 4a. In the spectra, the peaks of carbon (C), oxygen (O), nitrogen (N), and silicon (Si) atoms were observed due to the chemical structures of polymer blends and nanoclay. However, there is no peak of aluminum (Al). It was assessed that Al was not observed because it was located in the inner part of the nanoclay structure. The characteristic peak at 530 eV was assigned to the O 1s and that at 284 eV was assigned to the C 1s in both spectra of the nanocomposites. The peak of N 1s was observed at 398 and 397 eV for the Blend/Nanoclay-5 and Blend/Nanoclay-10, respectively. The presence of the Si 2p observed in the nanocomposites with the peak at 100 eV and Si 2s (at 152 eV for the Blend/Nanoclay-5 and 151 eV for the Blend/Nanoclay-10) peak in the XPS spectra indicates the presence of nanoclay in the nanocomposites and supports the formation of the nanocomposite.

**Figure 4:**
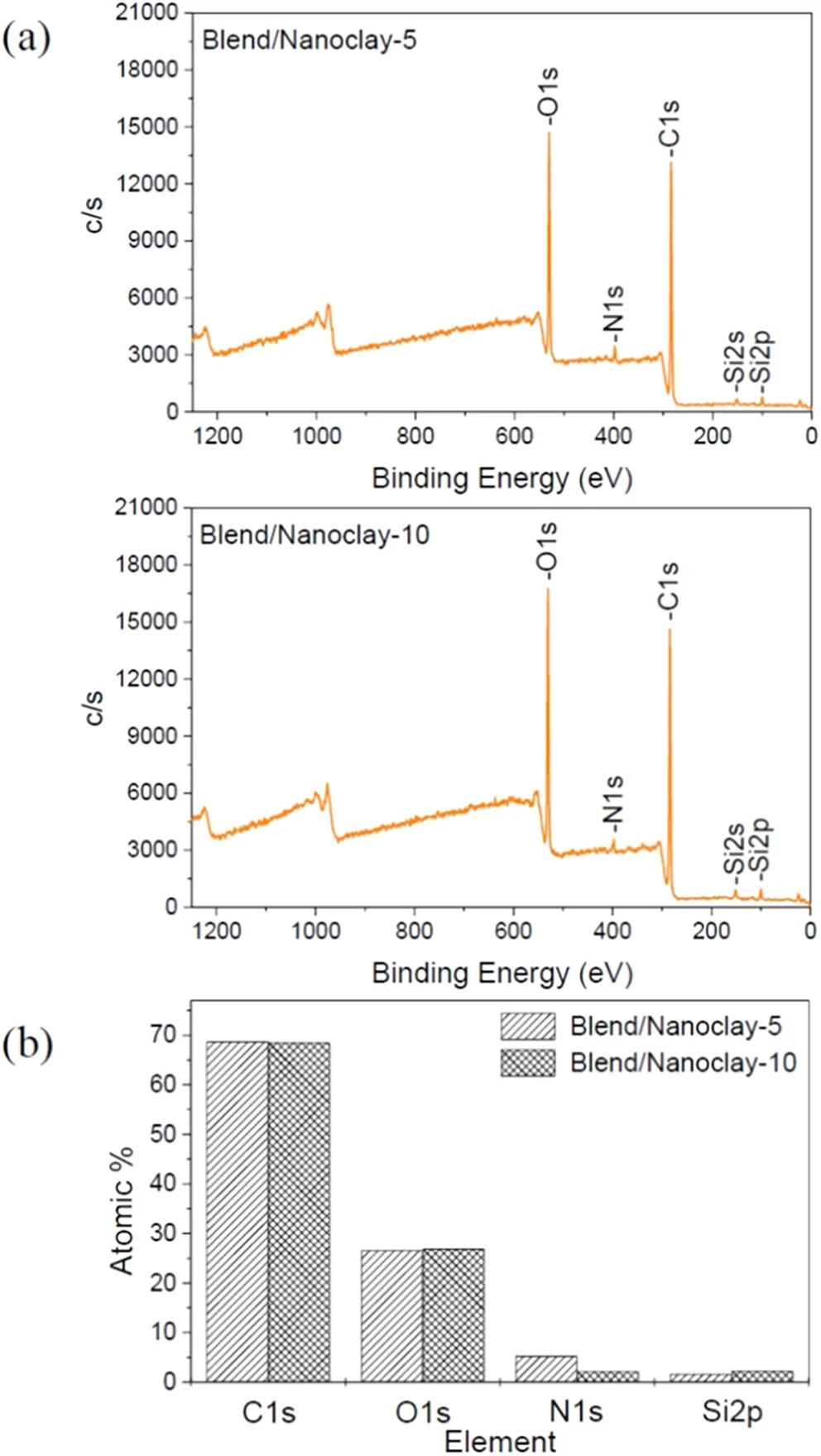
XPS survey spectra of the nanocomposites (a) and atomic % of the elements in the nanocomposites (b).

The atomic compositions of the nanocomposites were determined by an XPS analysis. The results are presented in Table 3 and Figure 4b. The relatively lower N 1s % in Blend/Nanoclay-10 can be interpreted as the PVP amount in the nanocomposite structure being less due to the high clay content. The atomic % of Si 2p in Blend/Nanoclay-10 appears to be higher than the atomic % of Si 2p in Blend/Nanoclay-5, in agreement with the preparation composition (Table 3). XPS analysis results revealed that nanocomposites were successfully prepared with supporting evidence of the presence of nanoclay.

**Table 3:** XPS Analysis Results of the Prepared Nanocomposites

	atomic % from survey scans				Si 2s		Si 2p	
nanocomposites	C 1s	O 1s	N 1s	Si 2p	BE (eV)	c/s	BE (eV)	c/s
Blend/Nanoclay-5	68.8	26.6	3.0	1.7	152	677.3	100	902.1
Blend/Nanoclay-10	68.6	26.9	2.2	2.3	151	790.7	100	971.9

TEM analysis is an important technique used to investigate the structures of nanocomposites (intercalated or exfoliated) by examining the distribution of nanoclays within the polymeric structure, and it supports the XRD results.^54^ In this work, TEM analysis was used to confirm the nanocomposite structures determined by the XRD analysis and to determine the nanoclay distribution in the ternary polymer blend system. While images obtained from TEM provide more localized information, results obtained from XRD provide information about the entire structure and were used as complementary techniques in determining the structures of the nanocomposites in this study. The TEM micrographs of the ternary polymer blend-based nanocomposites visualized the distribution of the nanoclay in the ternary polymer blend at a nanometer scale and are given at 50 and 100 nm magnifications (Figure 5). Low-magnification images show a general view of the dispersed nanoclay layers within the ternary polymer blend, whereas high-magnification images reveal the structures in more detail. The silicate layers can be seen as dark lines on the ternary polymer blend background, which appear as light gray in both nanocomposites. It has also been observed that they maintained their regular structure as seen in the images; this indicates that the obtained nanocomposites have a highly intercalated structure, which is indicated by the dashed orange circles in Figure 5. It can be thought that distribution can be achieved more easily at nanoclay percentages that are not too high (in Blend/Nanoclay-5). It is stated in the literature that polymer–clay nanocomposites containing less than 5% clay can generally be processed like polymers.^55^ It is seen that the homogeneous distribution of nanoclay is generally better in Blend/Nanoclay-5 than in Blend/Nanoclay-10. Additionally, a few partially exfoliated structures were observed in the micrograph of the Blend/Nanoclay-5 (shown in Figure 5, circled in blue in the 100 nm image). Although it is seen that dispersion is also achieved at Blend/Nanoclay-10, the presence of dark parts indicated by the purple arrow in Figure 5 shows the presence of aggregation resulting from a high percentage of nanoclay. Since the results obtained with TEM do not represent the entire material, images taken from different regions of the nanocomposite samples are shown in Figures S1–S5. Overall, these TEM analysis results supported the findings obtained from XRD analysis.

**Figure 5:**
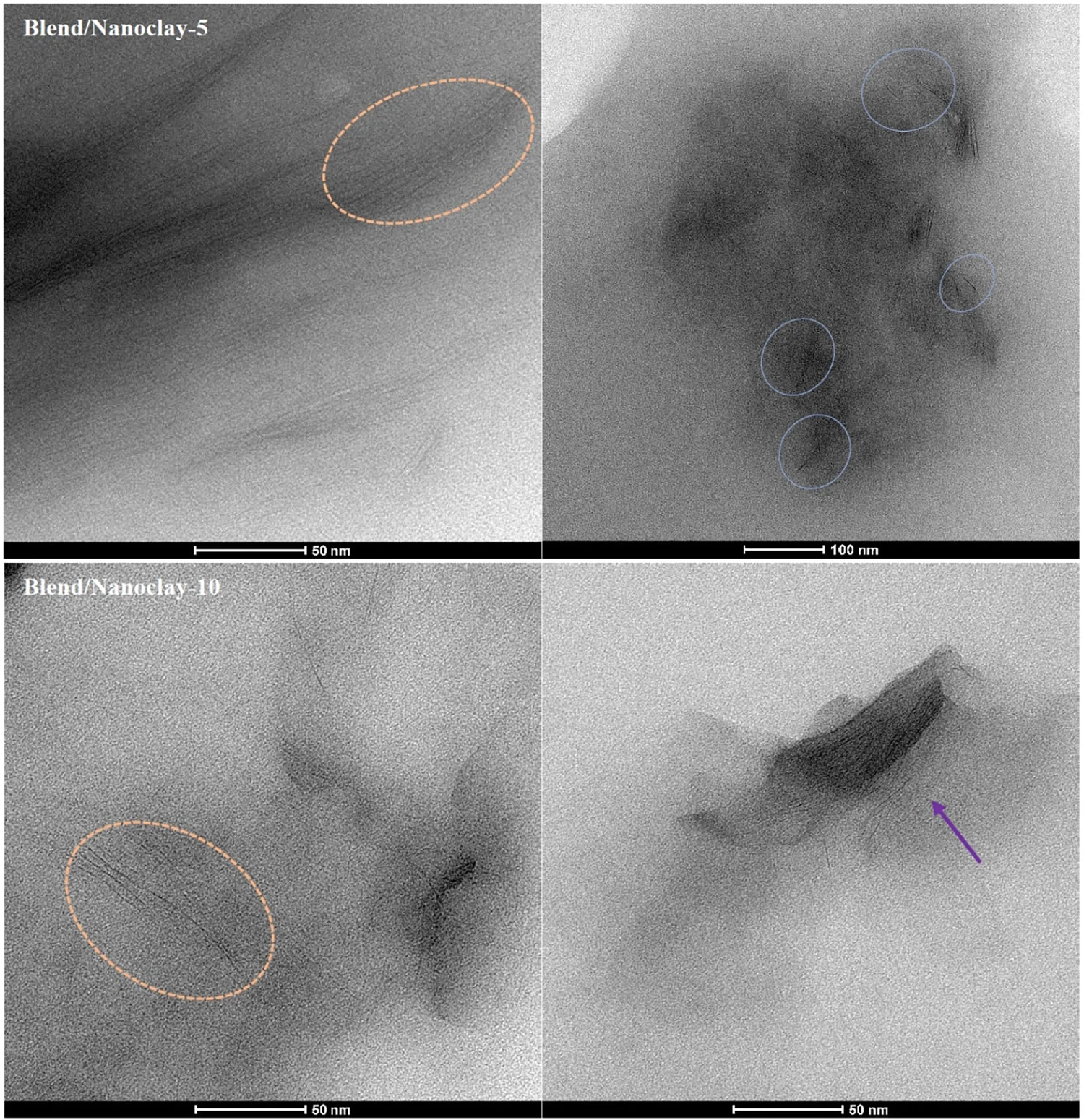
TEM micrographs of the nanocomposites at 50 and 100 nm magnifications.

### 3.2 Thermal Properties

TGA was used to investigate the effects of addition of nanoclay to the ternary polymer blend matrix on the thermal properties, thermal degradation, and thermal stability of the resulting nanocomposite materials. TGA thermograms of the polymeric materials are presented in Figure 6. It is seen that the thermal degradation of the prepared materials proceeds in more than one stage. Thermoanalytical results, temperature of the maximum mass loss rates for each stages, and mass loss % values of the prepared materials were determined from TG results at certain temperatures and presented in Tables 4 and 5.

**Figure 6:**
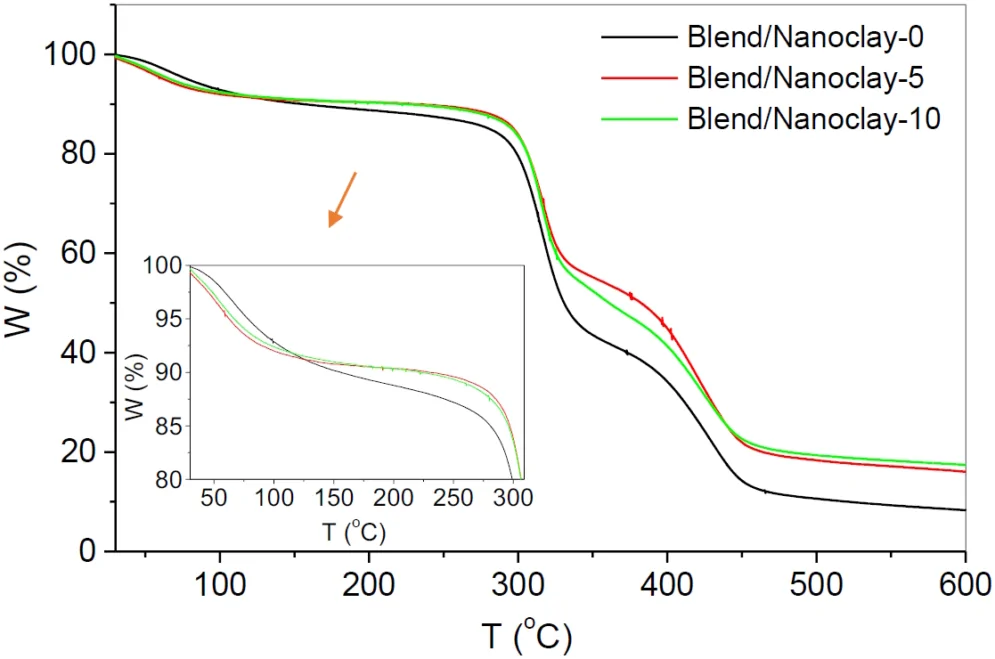
TGA curves of the materials.

**Table 4:** Thermoanalytical Results of the Materials

	mass loss % at certain *T* (°C)						
materials	100	200	300	400	500	600	residue at 600 °C (%)
Blend/Nanoclay-0	7.1	11.2	20.5	65.8	89.4	91.7	8.3
Blend/Nanoclay-5	8.0	9.6	16.2	55.2	81.6	83.9	16.1
Blend/Nanoclay-10	7.6	9.7	16.5	58.7	80.6	82.6	17.4

**Table 5:** TGA Results of the Materials

materials	*T*_m_ ^a^ (°C) 1st step	*T*_m_ ^a^ (°C) 2nd step	*T*_m_ ^a^ (°C) 3rd step	*T*_25_ ^b^ (°C)	*T*_50_ ^c^ (°C)	*T*_75_ ^d^ (°C)
Blend/Nanoclay-0	59.9	313.2	437.4	306.2	330.7	423.3
Blend/Nanoclay-5	57.0	317.0	424.9	312.1	382.1	440.7
Blend/Nanoclay-10	51.2	314.8	421.4	311.5	360.9	440.9

a Temperature of maximum mass loss rate for each step.

b Temperature at 25% mass loss.

c Temperature at 50% mass loss.

d Temperature at 75% mass loss.

The first stage resulted from water removal from ambient temperature to ∼100 °C in all samples and did not exceed 8%. Higher water loss was observed in Blend/Nanoclay-5 and Blend/Nanoclay-10 samples compared to the pristine ternary polymer blend. The adsorbed water molecules in the nanoclay structure can be found around metal ions in the exchangeable regions of the MMT.^56^ This could explain the higher percentage of water removal in nanocomposites containing nanoclay compared to the pristine ternary polymer blend.

A sharp change was observed in all materials at the second stage. The large drop in the TGA curves between ∼255 and 350 °C and the smaller third drop, starting from about 355 °C and continuing down to ∼485 °C, are observed due to the degradation and removal of polymeric organic units present, in the ternary polymer blend and long dimethyl dialkyl amine fragments in the nanocomposite structures. Interlayer water in organically modified layered silicate can also be removed at temperatures up to approximately 300 °C.^57^ It has been assessed that the degradation processes of polymeric units in this temperature range are as follows: in PVP, the removal of pyrrolidone units from the structure and the breaking of the main chain; in dextran, the decomposition of polysaccharide chains along with the breaking of glycosidic bonds and the opening of the pyranose ring; and in PEG, the formation of degradation products such as non-cyclic ethers and ethylene oxide through main chain degradation.^38,51,58^ In addition, Blend/Nanoclay-10 shows a very small decrease between the second and third steps at ∼357 °C, unlike the other two samples, and this difference is considered to be due to the removal of groups resulting from organic modification due to the high proportion of nanoclay in this sample.^57^ Thermal stabilities of nanocomposites were higher than the pristine polymer blend after nearly 125–600 °C temperature interval due to the addition of the nanoclay to the polymer blend structure. However, there was no significant difference in thermal stability with the addition of nanoclay at different amounts. This can be explained by the fact that the higher amount of nanoclay may not be uniformly distributed in the structure and agglomeration occurs with the increasing amount, and this can be seen in TEM results. In addition, the increase in dimethyl dialkyl amine organic groups caused by the surface modification due to the increasing amount of nanoclay can be considered another factor that reduces the thermal stability. The residue amount was 17.4 and 16.1% for Blend/Nanoclay-10 and Blend/Nanoclay-5, respectively, at 600 °C due to the thermal barrier effect of nanoclay. The lowest residue was 8.3% at this temperature for the pristine ternary polymer blend, as expected. This can be explained by the fact that after the polymeric units have degraded to a large extent, the increasing amount of nanoclay at high temperatures delays the mass transfer due to its barrier effect and thus increases the thermal stability.

The temperatures of the maximum mass loss rate (*T*_m_) for each step are shown in Table 5. This temperature can be determined from the derivative of the TG data. The highest point of the first derivative indicates the point on the weight loss curve where the greatest rate of change occurs. In the first stage, the *T*_m_ value in the water removal step was observed as 59.9, 57.0, and 51.2 °C for Blend/Nanoclay-0, Blend/Nanoclay-5, and Blend/Nanoclay-10, respectively. In the second step, the temperature of the maximum mass loss rate values of the nanocomposites were higher than the temperature of the maximum mass loss rate value of the pristine polymer blend, while they were lower in the third step. The temperature of the maximum mass loss rate ranges of the materials were observed as 313.2–317.0 and 421.4–437.4 °C for the all materials. These main decompositions come from the removal of the organic moieties due to the degradation of the functional groups in the polymer structure and modified sites of the nanoclay and the main chain ruptures of the polymeric units, as previously mentioned. The observed high thermal stability of the pristine polymer blend may be interpreted with the intermolecular interactions occurring in the blend and the possibility of physically cross-linked structure formation making the material stable at high temperatures. In the third step, unlike the second step, it was seen that the temperature of the maximum mass loss rate decreased remarkably with increasing nanoclay content.

When the temperatures at 25, 50, and 75% mass losses were analyzed, it was observed that the temperature values at which these mass losses were observed increased with the addition of organically modified nanoclay to the polymer blend. This result indicates that degradation is observed at higher temperatures because of the barrier effect of the added nanoclay. However, in the Blend/Nanoclay-10 nanocomposite containing 10% nanoclay, contrary to expectations, a decrease was observed compared to Blend/Nanoclay-5, but it was higher than that of the pristine polymer blend. It is thought that with the inclusion of a high amount of nanoclay in the structure (Blend/Nanoclay-10 nanocomposite), the nanoclay distribution may not be well-ordered compared to the nanocomposite containing a lower amount of nanoclay. In addition, as previously mentioned, large silicate aggregates may have formed locally in the structure containing a high percentage of nanoclay.

### 3.3 Dynamic Mechanical Properties

In dynamic mechanical analysis, sinusoidal oscillatory stress is applied to the prepared materials, leading to sinusoidal deformation. This applied oscillating stress is important in identifying the viscoelastic properties of the ternary polymer blend and ternary polymer blend-based nanomaterials. The dynamic mechanical behavior of all materials, including the relationships of storage modulus, loss modulus, and tan delta (tan δ) parameters with temperature, is shown in Figure 7. In the DMA curves of the pristine polymer blend, three main transitions were observed from all parameters. Storage modulus values were higher at the beginning and it decreased with increasing temperature step by step, as expected. The stiffness behavior of the polymer sample can be defined by storage modulus. Storage modulus describes the elastic response of the ternary polymer blend and ternary polymer blend-based nanoclay nanocomposites in these prepared systems. The onset temperature was observed at 47.8 °C from the storage modulus–temperature curve and storage modulus values continued to decrease with increasing temperature for the pristine ternary polymer blend. Second and third transitions were observed at 156.5 and 204.7 °C, respectively.

**Figure 7:**
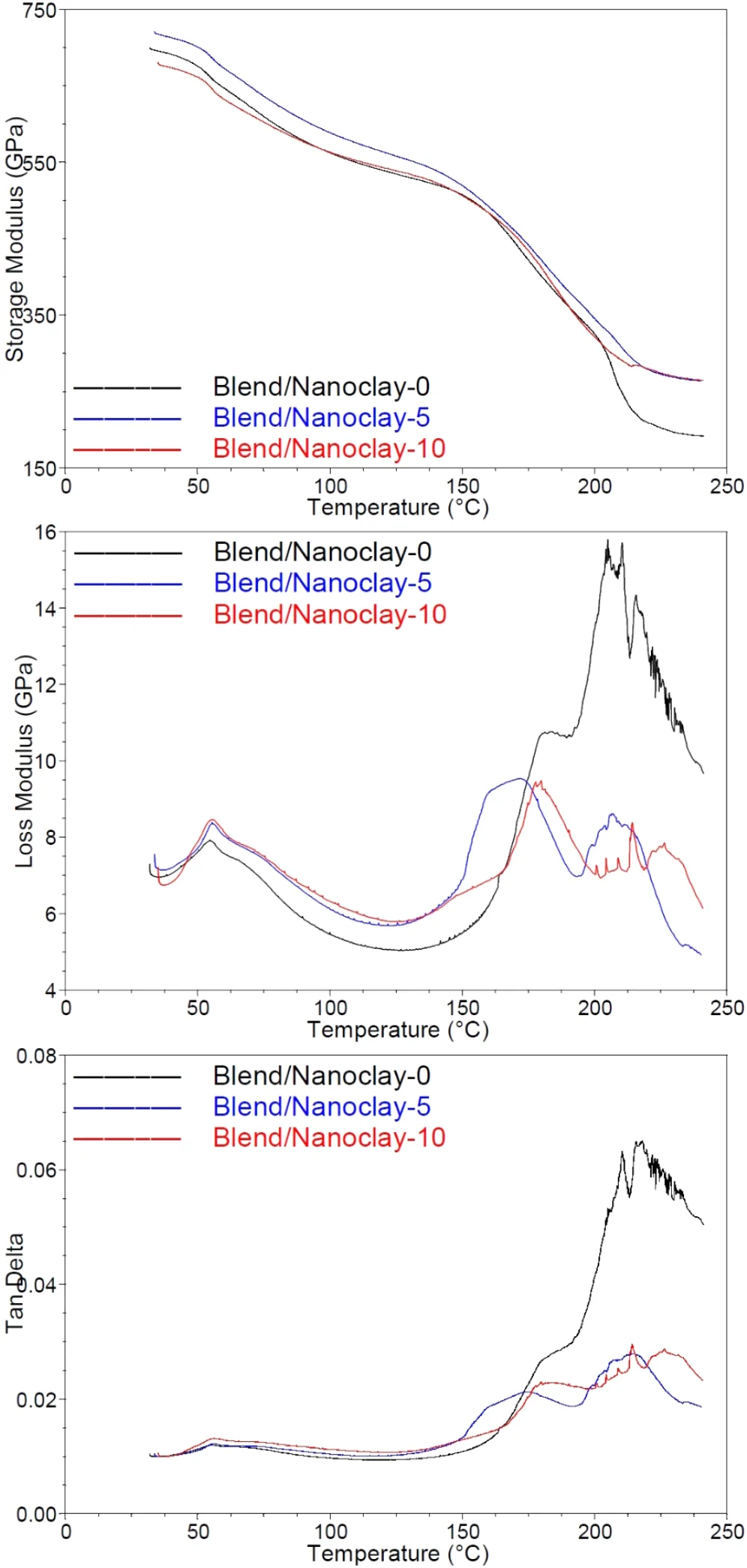
DMA curves of the materials.

Figure 8 shows the derivative storage modulus–temperature curves. To determine the inflection point precisely, the first derivative of the curve was plotted. Derivative curves can give more precise information about transition temperatures for the storage modulus–temperature curve. The minimum points in the derivative curves were seen at 54.5, 173.0, and 207.0 °C for the pristine polymer blend material. The first peak can be considered a response to a glass transition (α-transition). Other broadening and shouldered multiple peaks can be observed due to removal of water from the structure, the pseudo-melting behavior of the hydrogen-bonded structure, and the formation of some degradation products. The other two DMA parameters, loss modulus and tan δ, measure the dissipated energy and damping ability of the material, respectively. In the ternary polymer blend sample, the first maximum of the loss modulus and tan δ peaks at 54.6 °C associated with the glass transition is defined as α-relaxation. Relaxation is related to hindered rotations of the main polymer backbone. The observation of a broad single maximum peak indicates that the polymers in the prepared sample form a partially compatible blend. It shows that they coexist with intermolecular interactions by forming hydrogen bonds due to hydrogen bonding units in their structures.

**Figure 8:**
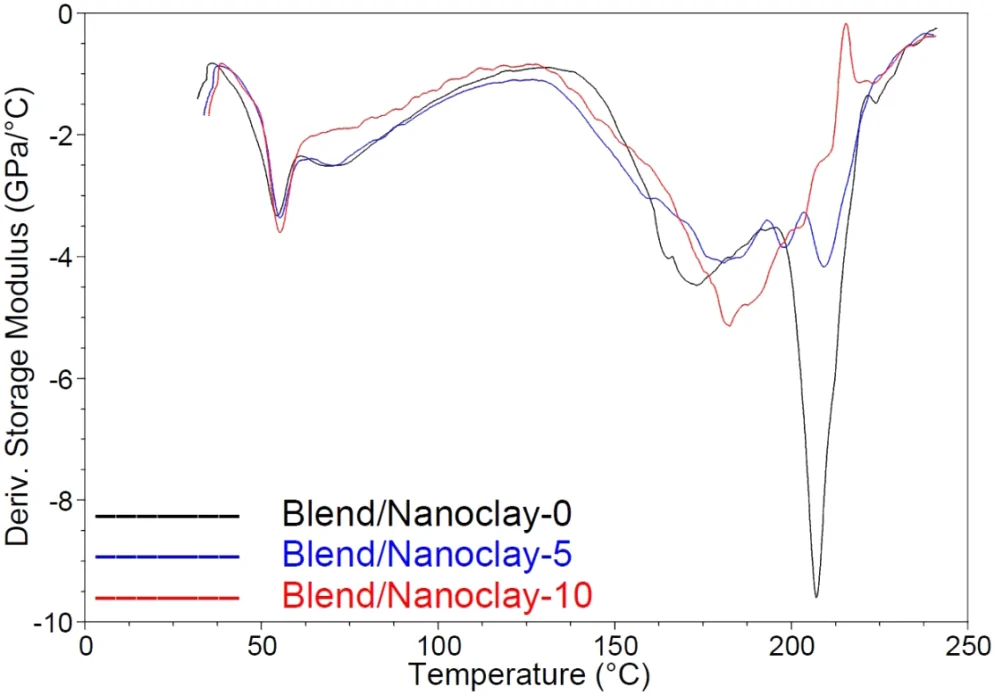
Derivative storage modulus–temperature curves of the materials.

Dynamic mechanical behavior of the nanocomposites is exhibited in Figure 7 in detail. The onset temperature of the storage modulus of Blend/Nanoclay-5 and Blend/Nanoclay-10 was calculated as 51.6 and 52.4 °C, for the first transition, respectively. These observed values are higher than that of the pristine polymer blend due to the presence of nanoclay in the nanocomposite structures. In addition, with the addition of nanoclay, an increase in the storage modulus of Blend/Nanoclay-5 was also observed, as expected.^59^ After the first transition, the second and third transitions were observed from the onset temperature of the storage modulus at 154.7 and 212.3 °C and 161.6 and 216.3 °C for the Blend/Nanoclay-5 and Blend/Nanoclay-10, respectively (Table 6). The derivative storage modulus curves of ternary polymer blend-based nanocomposites depending on temperature are seen in Figure 8. Minimum values of these curves were observed at 55.4, 181.0, and 209.4 °C for Blend/Nanoclay-5 and 55.4, 182.4, and 221.9 °C for Blend/Nanoclay-10. With the addition of nanoclay modified with organic groups to the pristine polymer blend, the interactions, which can be defined as the interfacial interactions occurring in the ternary polymer blend with functional groups, increased the temperatures with increasing nanoclay percentage. Besides, the limiting effect of rigid nanoclay particles on the chain movement of polymers also contributed to this increase.^60,61^

**Table 6:** DMA Results of the Materials^a^

	DMA parameters: *T*_1_, *T*_2_, *T*_3_ (°C)								
materials	*T*_1,o_ (SM)	*T*_2,o_ (SM)	*T*_3,o_ (SM)	*T*_1,m_ (LM)	*T*_2,m_ (LM)	*T*_3,m_ (LM)	*T*_1,m_ (tan δ)	*T*_2,m_ (tan δ)	*T*_3,m_ (tan δ)
Blend/Nanoclay-0	47.8	156.5	204.7	54.6	180.1	204.9	54.6	179.2	210.5
						210.4			215.7
						215.7			
Blend/Nanoclay-5	51.6	154.7	212.3	55.4	172.0	206.8	55.4	173.1	214.6
Blend/Nanoclay-10	52.4	161.6	216.3	55.3	179.9	214.2	55.4	179.9	214.2
						226.4			226.4

a SM: storage modulus; LM: loss modulus; tan δ: tan delta; o: onset; and m: maximum point.

Loss modulus and tan δ values were found from the maximum of the curves as 55.4, 172.0, and 206.8 °C and 55.4, 173.1, and 214.6 °C for the Blend/Nanoclay-5 and 55.3, 179.9, 214.2, and 226.4 °C and 55.4, 179.9, 214.2, and 226.4 °C for the Blend/Nanoclay-10, respectively (Table 6). The loss modulus and tan δ values increased with increasing temperature from the ambient temperature owing to the increase in the movements of the polymer chains until they reached a glass transition temperature (maximum point at the both curves) and then decreased due to energy loss. The first maxima observed in the loss modulus and tan delta curves of the polymer blend and nanocomposites were considered the glass transition temperatures of the materials, considering the known glass transition temperatures of the PVP, DEX, and PEG polymers that make up the ternary blend and ternary blend-based nanocomposites.^62,63^ Although the initial transitions show relatively broad peaks, the observation of a single peak is thought to indicate that the expected interaction in ternary polymer blends and nanocomposites has been achieved; however, it is also possible that multiple interactions and phase transitions are expected at similar temperatures, and furthermore, the broad peak in the polymer blend can also occur through limited miscibility. According to these results, when comparing the loss modulus and tan δ curves of polymer blends and nanocomposites, it was observed that the temperature values at which peak maxima for the first relaxation were seen in nanocomposites were slightly higher due to the intercalation of nanoclay. As the nanoclay ratio increased, no increase was observed due to the negative effects of high nanoclay percentage, such as agglomeration. Furthermore, it can be argued that water, which cannot be removed from the structure due to preparation conditions, may act as a plasticizer by lowering the glass transition temperature of the ternary polymer system and its nanocomposites.^64^ This effect also explains the decrease in the storage modulus curve that begins near the glass transition temperature. The final peak values, on the other hand, showed strong peak maxima at higher temperatures due to the removal of small volatile units such as dimethyl dialkyl amine in the modified nanoclay structure and the initiation of degradation.^48^ Furthermore, the second and third transitions observed at high temperatures may have occurred due to degradation products for materials containing different polymer structures as described in the TGA results.

## 4 Conclusions

The PVP/DEX/PEG ternary polymer blend and its nanocomposites containing 5 and 10% nanoclay were prepared with the solution mixing method. Structural, morphological, thermal, and dynamic mechanical behaviors of the prepared polymer blend and polymer blend nanocomposites containing different amounts of nanoclay were identified and exhibited. ATR-FTIR analyses revealed that the interactions between the ternary polymer blend containing functional groups and the nanoclay occur via hydrogen bonds. The strong Si–O stretching bands were observed at 1016 cm^–1^ due to the addition of nanoclay to the nanocomposite structure and the intensity of this band increased with increasing nanoclay percentage from 5 to 10% in the nanostructures. XRD analysis confirmed the intercalation of the ternary polymer blend to the silicate layers of nanoclay and formation of nanocomposite by changing *d*-spacing in the structures. Due to the increased nanoclay loading (5 and 10%) and the intercalation of the ternary polymer blend between the silicate layers, the XRD responses shifted to the lower 2θ region, and the *d*-spacing in the silicate layers of the ternary polymer blend-based nanocomposites increased to the range of 21.5–58.5%. The observation of peaks belonging to the Si in both nanocomposites in the XPS analysis data, and the proportionality of their atomic percentages to their composition, supports the conclusion that the nanocomposites were successfully obtained. The dispersion of the nanoclay in the ternary polymer blend and the morphology of the ternary polymer blend-based nanocomposites were also confirmed by the TEM analysis.

In general, the thermal stabilities of the ternary polymer blend-based nanocomposites, with the addition of nanoclay to the ternary polymer blend, were higher after about 100 °C compared to the pristine polymer blend. However, the addition of different percentages of nanoclay did not make a remarkable difference in thermal stability, since the high amount of nanoclay is not fully dispersed uniformly in the structure and agglomeration can occur with increasing amount. These results are also consistent with the XRD and TEM analysis. DMA results revealed that mostly small increases in glass transition temperatures were observed during the formation of nanocomposites via the intercalation process. However, in the nanocomposites, storage modulus onset temperatures began to decrease at higher temperatures due to the effect of the nanoclay. The fact that the nanocomposite containing 5% nanoclay has a higher storage modulus due to its highly intercalated structure and better dispersion of nanoclay at this percentage, and similarly, the decrease observed in the 10% nanoclay sample due to aggregation, is consistent with the other results.

Looking ahead, these findings present new opportunities for the development of nanosized materials with enhanced thermal properties, thanks to the functional groups in their structures, the synergies created by their combinations, and the nanoclay.

## Supplementary Material



## Data Availability

Data will be made available on request.

## Notes

Additional TEM images of nanocomposites (Figures S1–S5) (PDF)
