# Supplementary material for: Investigation of the Effect of Nanoclay on Thermal and Dynamic Mechanical Properties of Poly(N‑vinyl-2-pyrrolidone)/Dextran/Poly(ethylene glycol)-Based Nanocomposites
Source: ACS Omega. 2026 Sep 13;11(38):56993–7003. doi: 10.1021/acsomega.6c03988 (PMC13629362; doi:10.1021/acsomega.6c03988)
Supplement: Supplementary file 1 [file ao6c03988_si_001.pdf]

Supporting Information

Investigation of the Effect of Nanoclay on Thermal and Dynamic Mechanical Properties of Poly(*N*-vinyl-2-pyrrolidone)/Dextran/Poly(ethylene glycol)-Based Nanocomposites

Serap Kavlak*

Hacettepe University, Faculty of Science, Department of Chemistry, 06800 Ankara, Türkiye

***Correspondence to:** S. Kavlak (skavlak@hacettepe.edu.tr)

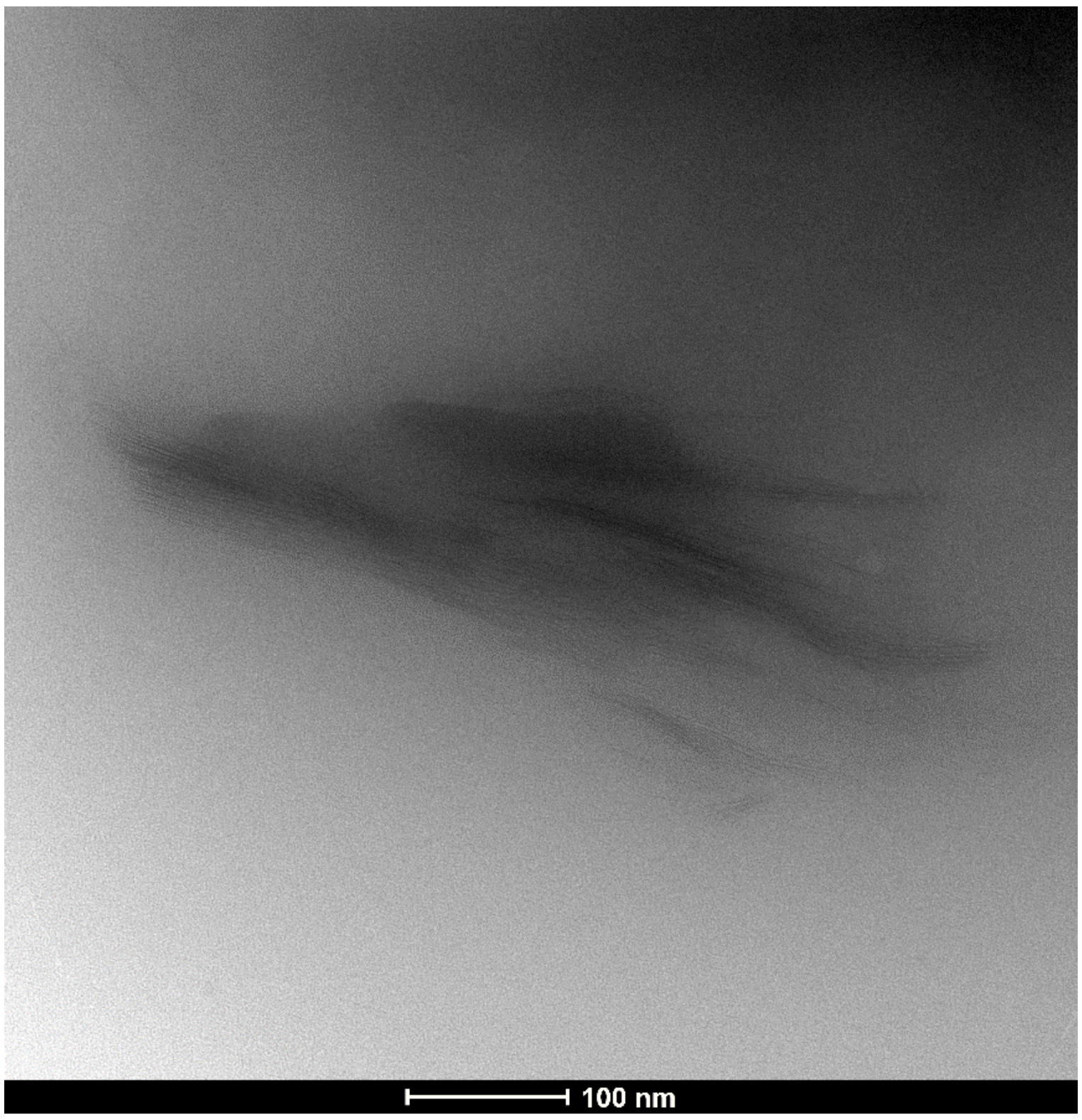

Figure S1. TEM micrograph of the prepared Blend/Nanoclay-5 at 100 nm magnification-1.

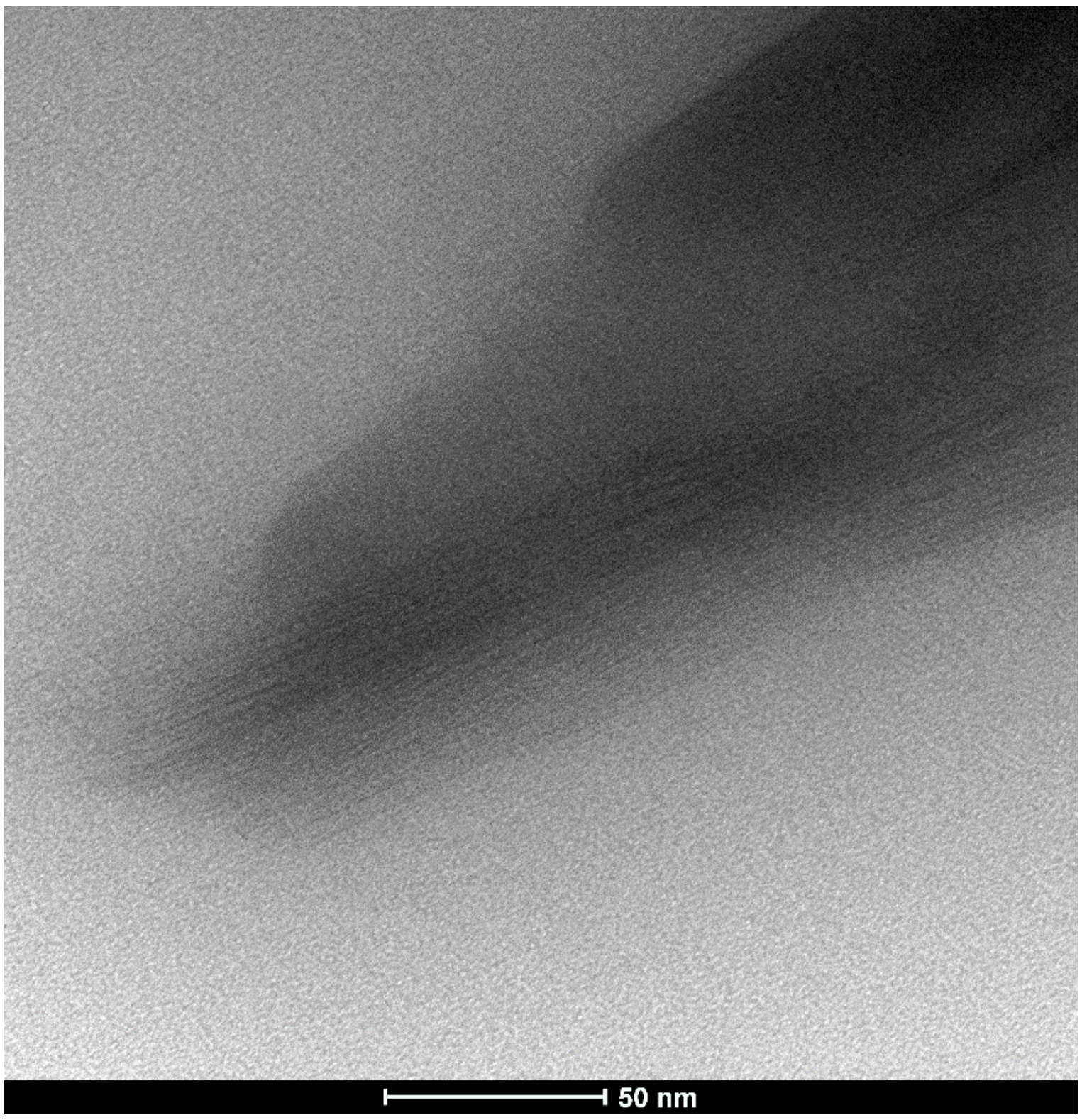

Figure S2. TEM micrograph of the prepared Blend/Nanoclay-5 at 50 nm magnification-2.

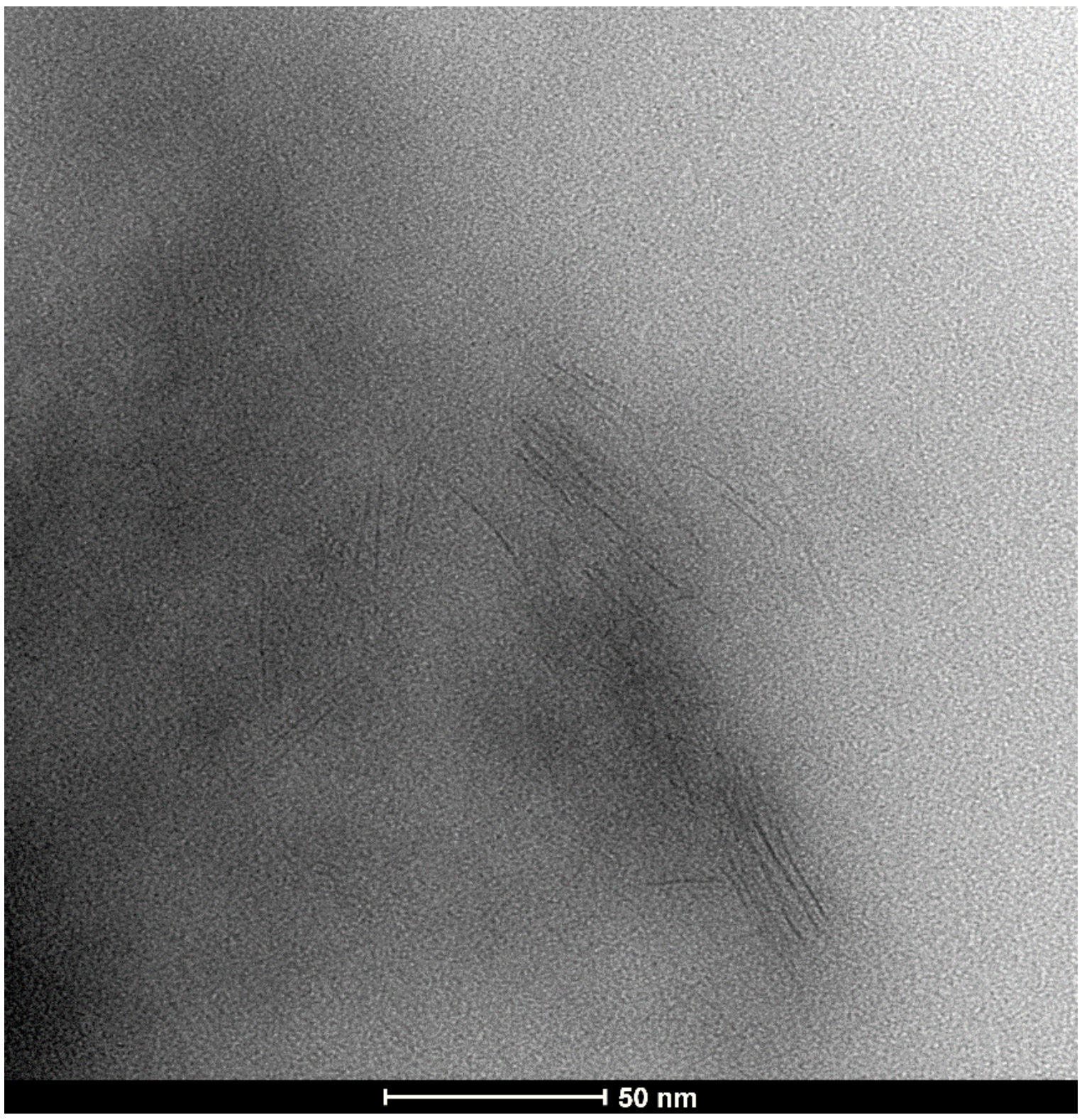

Figure S3. TEM micrograph of the prepared Blend/Nanoclay-5 at 50 nm magnification-3.

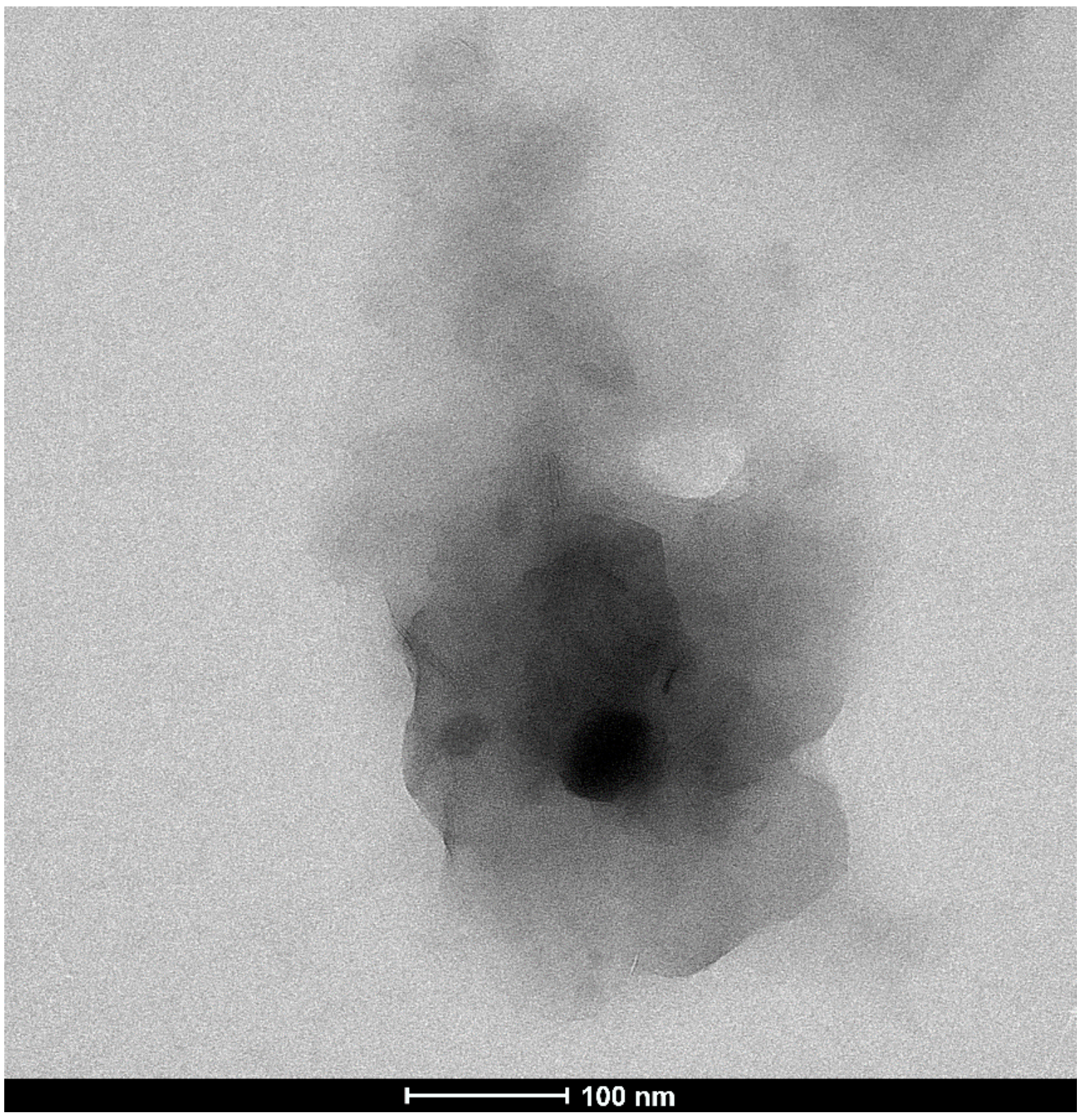

Figure S4. TEM micrograph of the prepared Blend/Nanoclay-10 at 100 nm magnification-1.

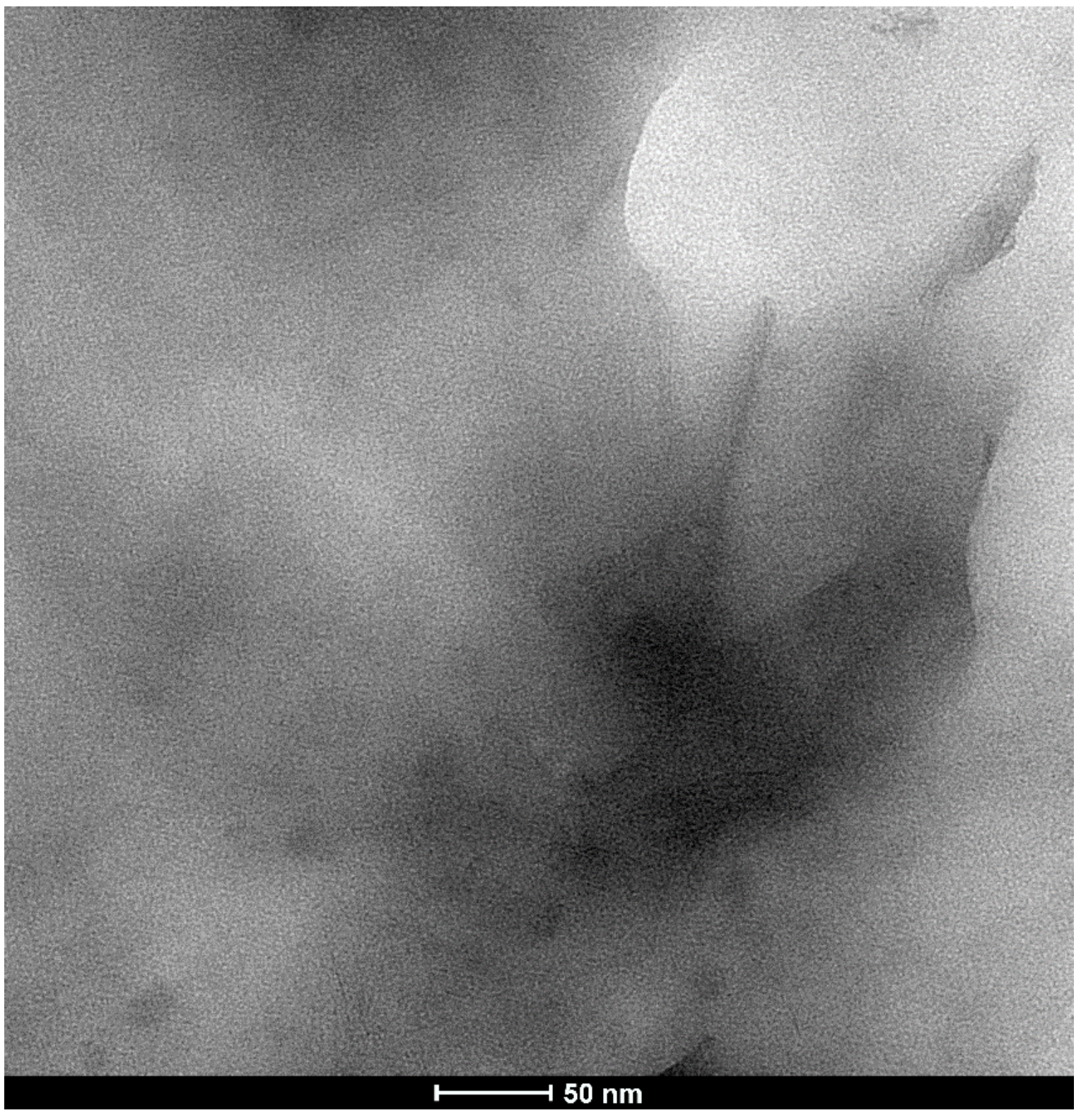

Figure S5. TEM micrograph of the prepared Blend/Nanoclay-10 at 50 nm magnification-2.
